# *In Vitro* Protective Effects of a Standardized Extract From *Cynara Cardunculus* L. Leaves Against TNF-α-Induced Intestinal Inflammation

**DOI:** 10.3389/fphar.2022.809938

**Published:** 2022-02-11

**Authors:** Antonio Speciale, Claudia Muscarà, Maria Sofia Molonia, Giovanni Toscano, Francesco Cimino, Antonella Saija

**Affiliations:** Department of Chemical, Biological, Pharmaceutical and Environmental Sciences, University of Messina, Messina, Italy

**Keywords:** inflammatory bowel disease, Cynara cardunculus, Caco-2, Nrf2, oxidative stress

## Abstract

Inflammatory bowel disease (IBD) represents a group of progressive disorders characterized by recurrent chronic inflammation of the gut. New unconventional therapies based on plant derived compounds capable of preventing and/or reducing acute or chronic inflammation could represent a valid alternative for the treatment or prevention of IBDs. *Cynara cardunculus* L. leaves, considered a food-waste suitable as a rich source of bioactive polyphenols including luteolin and chlorogenic acid, has been reported for its positive effects in digestive tract. The aim of the present work was to evaluate the *in vitro* molecular mechanisms of beneficial effects of a standardized polyphenol-rich extract obtained from the leaves of *Cynara cardunculus* L (CCLE) against acute intestinal inflammation induced by TNF-α on intestinal epithelial Caco-2 cells. CCLE prevented TNF-α-induced NF-κB inflammatory pathway and the overexpression of IL-8 and COX-2. In addition, CCLE was able to improve basal intracellular antioxidant power in both TNF-α-unexposed or -exposed Caco-2 cells and this effect was associated to the activation of Nrf2 pathway, a master regulator of redox homeostasis affecting antioxidant and phase II detoxifying genes, stimulating an adaptive cellular response. In conclusion, our data clearly evidenced that, although considered a waste, *Cynara cardunculus* leaves may be used to obtain extracts rich in bioactive polyphenols potentially useful for prevention and treatment of inflammatory intestinal diseases.

## Introduction

Chronic inflammatory intestinal disorders, also known as inflammatory bowel diseases (IBDs), including Crohn’s disease and ulcerative colitis, are a group of conditions that affect the colon and small intestine. These diseases are characterized by chronic immune-mediated inflammation and progressive alteration of the gastrointestinal tract (Michielan and D'Incà, 2015). Many studies have been carried out to identify the etiology and pathogenesis of IBDs. Currently, the most accredited hypothesis is that, in genetically predisposed subjects, a series of environmental stimulations and/or the intestinal flora itself can determine the loss of inflammation control mechanisms in the gut, with consequent activation of the local immune response and amplified production of proinflammatory cytokines (Sobczak et al., 2014). The cytokine tumor necrosis factor-α (TNF-α) plays a critical role in the pathogenesis of IBDs and its levels are significantly increased in IBDs patients both in the intestinal mucosa (Ngo et al., 2010) and in the serum (Thomson et al., 2012). It triggers the activation of the nuclear transcription factor kappaB (NF-κB), a key regulator involved in inflammatory processes, promoting the expression of various proinflammatory genes (Atreya et al., 2008). In addition, oxidative stress is one of the most important etiological and/or triggering factors in IBDs since reactive oxygen species (ROS) have been associated with the onset and progression of IBDs (Cross and Wilson, 2003). In fact, ROS are involved in the up-regulation of various genes involved in the adaptive and innate immune response within the gastrointestinal tract (Biasi et al., 2013), and they act as modulators of redox-sensitive transcription factors, such as NF-κB and Nuclear factor-2 erythroid related factor-2 (Nrf2) (Sakon et al., 2003). NF-κB is an important regulator of physiological processes at the level of the intestinal epithelium, by modulating the immune homeostasis of intestinal epithelial cells and the permeability of the intestinal barrier (Pasparakis, 2008). However, ROS-induced hyperactivation of NF-κB causes an increase in the transcription of genes encoding pro-inflammatory cytokines such as IL-1, IL-6, IL-8, IL-16, TNF-α (Pallone and Monteleone, 2001; Lingappan, 2018) and an increase in intestinal permeability (Al-Sadi et al., 2016). On the contrary, Nrf2 transcription factor regulates the expression of phase II detoxifying and antioxidant enzyme genes including those codifying for NAD(P)H quinone oxidoreductase 1 (NQO1), heme oxygenase-1 (HO-1), and Glutamate-Cysteine Ligase Catalytic Subunit (GCLC), and thus plays an important role in cellular defense against cellular injury caused by oxidants and ROS (Speciale et al., 2011b). Moreover, a crosstalk between the NF-κB and Nrf2 signaling pathways has been hypothesized by several authors (Ahmed et al., 2017; Bellezza et al., 2018; Speciale et al., 2020), useful in regulating the fine balance of cellular redox status and responses to stress and inflammation (Saha et al., 2020).

Currently, conventional IBDs therapies include aminosalicylates and corticosteroids, generally indicated for mild to moderate conditions, and immunosuppressive agents used in moderate to severe clinical conditions. Unfortunately, these drugs often cause considerable side effects and therapeutic failure. Therefore, new unconventional therapies based on plant derived compounds capable of preventing and/or reducing acute or chronic inflammation could represent a valid alternative for the treatment or prevention of IBDs.

Polyphenols are plant secondary metabolites, contained in numerous edible fruits and vegetables (D'Archivio et al., 2007), exhibiting antioxidant, immunomodulatory and anti-inflammatory properties (Kaulmann and Bohn, 2016; Le Sage et al., 2017). In particular, polyphenols have shown beneficial effects in intestinal diseases (Nunes et al., 2018; Li et al., 2020), so that they could be taken into consideration as new therapeutic agents in the prevention and treatment of IBDs. *Cynara cardunculus* (L.) is an herbaceous plant belonging to the Asteraceae family, usually cultivated in Mediterranean countries, and is abundantly used in human nutrition (Lattanzio et al., 2009). Its use has very ancient origins and it has been defined as a drug in traditional medicine thanks to its positive action in digestive function, in diseases of the biliary tract, in anemia and in atherosclerosis (Gebhardt and Fausel, 1997; Kraft, 1997). In particular, *Cynara cardunculus* leaves are rich in numerous polyphenols, including luteolin, chlorogenic acid, and cynarin, which are the active substances more involved in the protective antioxidant and anti-inflammatory activity (Zayed et al., 2020). Many studies have reported that extracts obtained from the leaves of *Cynara cardunculus* (L.) have beneficial properties for human health thanks to hypoglycemic, hypercholesteremic, hepatoprotective, anti-atherosclerotic antioxidant, prebiotic and probiotic, choleretic, anti-inflammatory, cardiovascular health and digestive effects (Ben Salem et al., 2015). Furthermore, these extracts have been shown to be well tolerated and widely safe for humans, and therefore could be used as complementary approaches to existing conventional therapies (Ben Salem et al., 2015).

In addition, since only the immature composite inflorescence and the receptacle are the edible part of this plant, at the end of the crop cycle, leaves represent a very abundant byproduct suitable as a rich source of bioactive compounds, including phenolic compounds (Pagano et al., 2016). Thus, the aim of the present work was to evaluate the molecular mechanisms of the *in vitro* beneficial effects of a standardized polyphenol-rich extract obtained from the leaves of *Cynara cardunculus* L. subsp. *Scolymus* Hayek against acute intestinal inflammation induced by TNF-α on intestinal epithelial Caco-2 cells. In particular, we studied the anti-inflammatory and antioxidant effects focussing on the modulation of the NF-κB and Nrf2 signaling pathways.

## Materials and Methods

### Reagents

DMSO was acquired by AppliChem (Darmstadt, Germany). TNF-α was purchased from PeproTech Inc. (Rocky Hill, NJ, United States). All other reagents, unless otherwise specified, were acquired from Sigma-Aldrich (Milan, Italy). In our study we used a commercially available standardized extract (Altilix^®^, Bionap Srl, Italy) obtained from leaves of *Cynara cardunculus* (L.) [Asteraceae] (CCLE) rich in chlorogenic acid and luteolin (10–12% chlorogenic acid and derivates, and 2–4% luteolin-7-glucoside and derivates).

### Cell Culture and Treatments

Caco-2 epithelial cells, obtained from ATCC, were grown in DMEM supplemented with 10% FBS, 4 mM L-glutamine, 1% non-essential amino acids, 100 U/mL penicillin, and 100 μg/ml streptomycin. Cells were maintained at 37°C in a humidified atmosphere with 95% air and 5% CO_2_. Caco-2 monolayers were prepared by seeding cells at 4 × 10^4^ per cm^2^ on the upper side of transwell inserts (0.4 µm pore size; Greiner Bio-One, Italy) and cultured for 18 days postconfluence to obtain fully differentiated cells (Ferrari et al., 2016; Ferrari et al., 2017). Monolayer integrity and formation of tight junction (TJ) were assessed by measurement of Trans-Epithelial Electrical Resistance (TEER) by using a Millicell-ERS Voltohmmeter (Millipore, MA, United States ). Monolayers used in this study had TEER values ≥600 Ω x cm^2^.

Differentiated Caco-2 monolayers, prepared as above described, were pretreated or not with CCLE (5-10-15 μg/ml) for 24 h added only to the apical compartment of the transwell inserts. CCLE was always freshly dissolved in DMSO and immediately used. For all experiments, the final concentration of DMSO in the culture medium was always ≤ 0.1% (*v/v*). After 24 h, cells were washed twice with DPBS and then exposed for 6 h to 50 ng/ml TNF-α added in both the apical and the basolateral compartments of the transwell inserts. TNF-α concentration was chosen on the basis of preliminary experiments indicating that exposure to 50 ng/ml significantly decreased TEER value already after 3 h compared to the untreated control cells (Ferrari et al., 2016).

### Cell Lysates Extraction

Following the appropriate treatment, whole cell lysate was prepared in non-denaturing lysis buffer (10 mM Tris HCl, pH 7.4, 150 mM NaCl, 1% Triton X-100, and 5 mM EDTANa_2_) containing protease inhibitors (1 μg/ml leupeptin, 1 mM benzamidine, 2 μg/ml aprotinin) and 1 mM DTT. Nuclear and cytoplasmic extracts were prepared as described elsewhere (Cimino et al., 2013). All the protein fractions were stored at −80°C until use. Protein concentration in lysates was determined using the Bradford reagent (Bradford, 1976), using bovine serum albumin (BSA) as standard.

### Western Blot Analysis

For immunoblot analyses, 20 μg of protein lysates per sample were denatured in 4 × SDS-PAGE sample buffer (260 mM Tris–HCl, pH 8.0, 40% (*v/v*) glycerol, 9.2% (*w/v*) SDS, 0.04% bromophenol blue and 2-mercaptoethanol as reducing agent) and subjected to SDS-PAGE on 10% acrylamide/bisacrylamide gels. In order to determine p65 and Nrf2 nuclear level, nuclear lysates were used; COX-2 levels were determined in whole cell lysates, whereas pIKK α/β in cytoplasmic lysates. Separated proteins were transferred to PVDF membrane (Hybond-P PVDF, Amersham Bioscience). Residual binding sites on the membrane were blocked by incubation in TBST (10 mM Tris, 100 mM NaCl, 0.1% Tween 20) with 5% (w/v) nonfat milk powder for 3 h at room temperature. Membranes were then probed with specific primary antibodies: rabbit anti-NF-kB p65 polyclonal antibody (Thermo Fisher Scientific) (1:1,000); rabbit anti-Nrf2 polyclonal antibody (Santa Cruz Biotechnology) (1:200); rabbit anti-Phospho-IKK α/β (Ser176/180) monoclonal antibody (Cell Signaling Technology) (1:1,000), mouse anti-COX-2 monoclonal antibody (Santa Cruz Biotechnology) (1:200); rabbit anti-β-Actin monoclonal antibody (Cell Signaling Technology) (1:6,000), rabbit anti-Lamin-B monoclonal antibody (Cell Signaling Technology) (1:1,500), followed by peroxidase-conjugated secondary antibody: anti-rabbit Ig (Cell Signalling Technology) (1:6,000) and anti-mouse Ig (Cell signalling Technology) (1:10,000), visualized with an ECL plus detection system (Amersham Biosciences). The equivalent loading of proteins in each well was confirmed by Ponceau staining and β-actin or Lamin B control. Quantitative analysis was performed by densitometry.

### Real-Time PCR

RNA was extracted using the E. Z.N.A. Total RNA Kit I am following manufacturer’s instructions (OMEGA Bio-Tek VWR), quantified with Quant-iT™ RNA assay kit by QUBIT fluorometer (Invitrogen, Milan, Italy), and reverse transcripted with the M-MLV reverse transcriptase. 7,300 Real-Time PCR System (Applied Biosystems, Monza, Italy) coupled with the Sybr green JumpStart™ Taq Ready Mix kit was used for the assessment of gene expression. The specific primers used were: 18S rRNA, forward, 5′-GTA ACC CGT TGA ACC CCA TT-3′, reverse, 5′-CCA TCC AAT CGG TAG TAG CG-3’; IL-8, forward, 5′-ACT GAG AGT GAT TGA GAG TGG AC-3′, reverse, 5′-AAC CCT CTG CAC CCA GTT TTC-3’ (Ferrari et al., 2017); GCLC, forward, 5′-GGC ACA AGG ACG TTC TCA AGT-3′, reverse, 5′-CAG ACA GGA CCA ACC GGA C-3’ (Primer Bank ID: 308199422c2) (Wang and Seed, 2003); NQO1, forward, 5′-AAG AGC ACT GAT CGT ACT GG-3′, reverse, 5′- CTT CAG TTT ACC TGT GAT GTC C-3’ (Speciale et al., 2011a). Cycling conditions were 40 cycles of 94°C denaturation (15 s), 60°C annealing and extension (1 min). The fold increase of mRNA expression, compared with the control cells not pretreated and not exposed to TNF-α, and corrected with 18S rRNA housekeeping gene, was determined using the 2^−ΔΔCt^ method (Schmittgen and Livak, 2008).

### Intracellular Total Antioxidant Activity (TAA)

Following the appropriate treatment, cells were collected and then homogenized. The ability to scavenge the 2,2′-azinobis-3-ethylbenzothiazine-6-sulfonic acid (ABTS+) radical was evaluated. Briefly, this method determines the capacity of antioxidants to quench the ABTS + radical. The ABTS + radical cation was produced as elsewhere described (Dehimi et al., 2016). Either sample or Trolox standard solution were added to 2 ml of ABTS radical solution, and the absorbance was recorded at 734 nm with an UV–vis spectrophotometer (Shimadzu, Japan) after allowing the reaction to stand for 6 min in the dark at room temperature. Each determination was performed in triplicate and repeated three times. Results were expressed as nmoles of trolox equivalents/mg of proteins (Fratantonio et al., 2017) using a trolox standard calibration curve.

### Intracellular Levels of Glutathione

The intracellular concentration of reduced glutathione (GSH) was detected by a uHPLC LC-30AD Nexera equipped with a RF-20A Prominence fluorescence detector and an automatic SIL 30-AC Nexera sample injector (Shimadzu, Milan, Italy). Briefly, cells were lysed in 90 µl of cold water by three cycles of freezing-thawing and sonication for 3 min, centrifuged, and the supernatant was collected and used to determine the content of GSH by HPLC analysis of the GSH-*o*-phthalaldehyde (OPA) adducts. To determine GSH, the proteins in the supernatant (40 µl) were precipitated with 85 µl of 2.5% 5-sulfosalicylic acid (SSA, *w/v*) and centrifuged; then, the deproteinized supernatant was used directly for OPA derivatization. Derivatization with OPA was performed at room temperature by mixing 100 µl of the deproteinized supernatant with 100 µl of OPA (5 mg/ml). After 1 min, the samples were neutralized and diluted by the addition 500 µl of 100 mM sodium phosphate at pH 7.0. As previously reported (Trombetta et al., 2010), an isocratic eluent program was utilized with 7,5 % of CH_3_OH and 92,5 % acetate buffer at pH 7,00±0.1 over a C18 Nucleodur Gravity 3 µm column and a Nucleosil guard column both Macherey-Nagel GmbH (Dueren, Germany). The fluorescence parameters were λex = 340 nm and λem = 420 nm. Under these conditions the GSH derivatized adduct (as below descripted) was eluted at 12,1 retention time. A standard curve for the glutathione derivative was obtained up to 100 μM; a linear regression was obtained with a *r* ^2^ = 0,995. Results are normalized with the protein content determined by using the Bradford assay (Bradford, 1976).

### ROS Measurement by Dichlorodihydro-Fluorescein Diacetate Assay

Generation of ROS was measured by the oxidation-sensitive fluorescent probe, dichloro-dihydro-fluorescein diacetate (DCFH-DA), as a modified method previously described (Fratantonio et al., 2017). Briefly, at the end of 6 h exposure to TNF-α, cells were washed three times with DPBS (without Ca^2+^ and Mg^2+^) at both the compartments and treated with DCFH-DA 50 μM in the apical side at 37°C for 30 min in the dark. DCFH-DA was then removed, and cells were washed three times with DPBS (pH 7.4) to remove excess probe. Then, fluorescence was determined at 485 and 530 nm (excitation and emission, respectively) using a fluorimeter. The fluorescence intensity is directly proportional to the ROS amount. ROS levels were expressed as DCFH-DA relative fluorescence intensity and reported as percentage of control. Each analysis was carried out in triplicate.

### Statistical Analysis

All the experiments were performed in triplicate and repeated three times. Results are expressed as mean ± SD from three experiments and statistically analyzed by a one-way or a two-way ANOVA test, followed by Tukey’s HSD, using the statistical software ezANOVA (http://www.sph.sc.edu/comd/rorden/ezanova/home.html). Differences in groups and treatments were considered significant for *p* < 0.05.

## Results

### Protective Effect of CCLE on TNF-α-Induced NF-κB Pathway Activation

NF-κB is a widely expressed pleiotropic transcriptional factor, and it is the master regulator involved in the transcription of genes that modulate inflammation. The most common form of active NF-κB in humans is a heterodimer composed of p65 and p50 subunits (Bernotti et al., 2003), and p65 subunit is able to activate the transcription of proinflammatory target genes. In baseline conditions, inactive NF-κB dimers reside in the cytoplasm retained by binding to IκBs inhibitory proteins. As a result of different stimuli involving LPS, viruses, and proinflammatory cytokines such as IL-1 or TNF-α, the IKK complex (composed of the catalytic subunits IKKα, IKKβ and the regulatory subunit IKKγ) is activated (Karin and Greten, 2005). The active IKK complex, phosphorylating IκBs bonded to NF-κB, releases the NF-κB dimers that translocate into the nucleus and modulate the transcription of target genes (Hayden and Ghosh, 2004).

**FIGURE 1:**
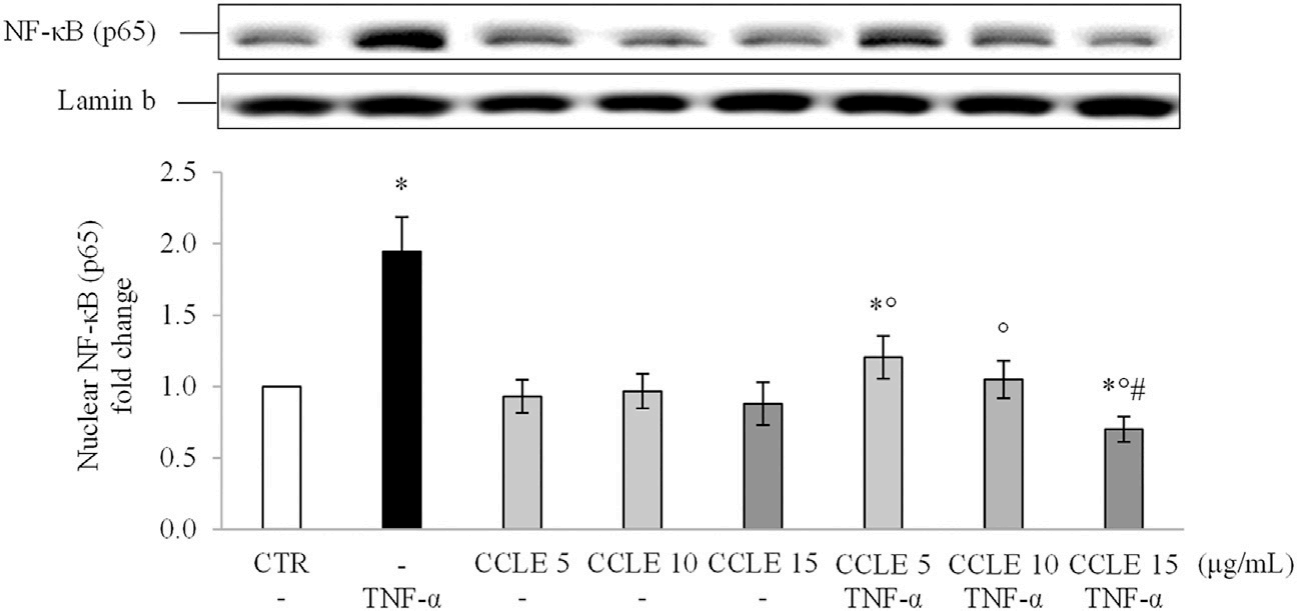
Nuclear NF-κB (p65). The Caco-2 monolayer was pretreated with CCLE (5, 10, and 15 μg/ml) for 24 h, and subsequently exposed to 50 ng/ml TNF-α for 6 h. Cultures treated with the vehicles alone were used as controls (CTR). Caco-2 nuclear lysates were analysed by Western blot, and nuclear localization of the p65 protein was evaluated. NF-κB (p65) intensity values were normalized to the corresponding Lamin B values. Results are reported as fold change against CTR and expressed as mean ± SD of three independent experiments. ^*^ *p* < .05 vs CTR; °*p* < .05 vs TNF-α; ^#^ *p* < .05 vs lower doses of CCLE + TNF-α.

To understand the effect of CCLE on the inflammatory response, the activation of the proinflammatory NF-κB pathway was evaluated by determination of the p65 nuclear location through Wester blot. Exposure to TNF-α triggered activation of NF-κB pathway as observed by high p65 levels in the nuclear fraction of Caco-2 cells exposed to TNF-α 50 ng/ml (Figure 1). Pretreatment with CCLE significantly suppressed activation of the inflammatory pathway induced by TNF-α, reducing p65 nuclear levels to values measured in control cells. Interestingly, cells exposed to CCLE 15 μg/ml showed significantly lower NF-κB nuclear levels than those of control cells. Pretreatment with CCLE alone did not affect nuclear p65 levels.

**FIGURE 2:**
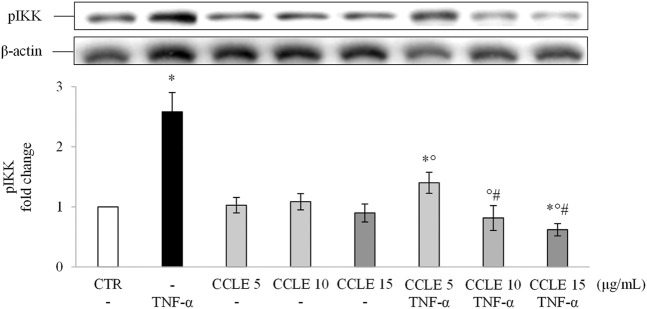
pIKK α/β. The Caco-2 monolayer was pretreated with CCLE (5, 10, and 15 μg/ml) for 24 h, and subsequently exposed to 50 ng/ml TNF-α for 6 h. Cultures treated with the vehicles alone were used as controls (CTR). pIKK α/β intensity values were normalized to the corresponding β-actin value. Results are reported as fold change against CTR and expressed as mean ± SD of three independent experiments. ^*^ *p* < .05 vs CTR; *°p* < .05 vs TNF-α; ^#^ *p* < .05 vs CCLE 5 μg/ml + TNF-α.

Since NF-κB translocation depends on the IKK complex, the effect of CCLE on phosphorylation (activation) of this complex has been evaluated to determine whether CCLE reduces p65 nuclear accumulation by inhibition of IKK. As shown in Figure 2, the pretreatment with CCLE suppressed the activation/phosphorylation of IKKα/β induced by TNF-α, obtaining, already with the concentration 10 μg/ml, values similar to those found in control cells. Also in this case, CCLE 15 μg/ml reduced basal levels of pIKK. Therefore, these data confirm the inhibitory effect of CCLE on NF-κB pathway induced by TNF-α through inhibition of IKK activation.

### CCLE Modulates IL-8 Gene Expression Induced by NF-κB Pathway Activation

**FIGURE 3:**
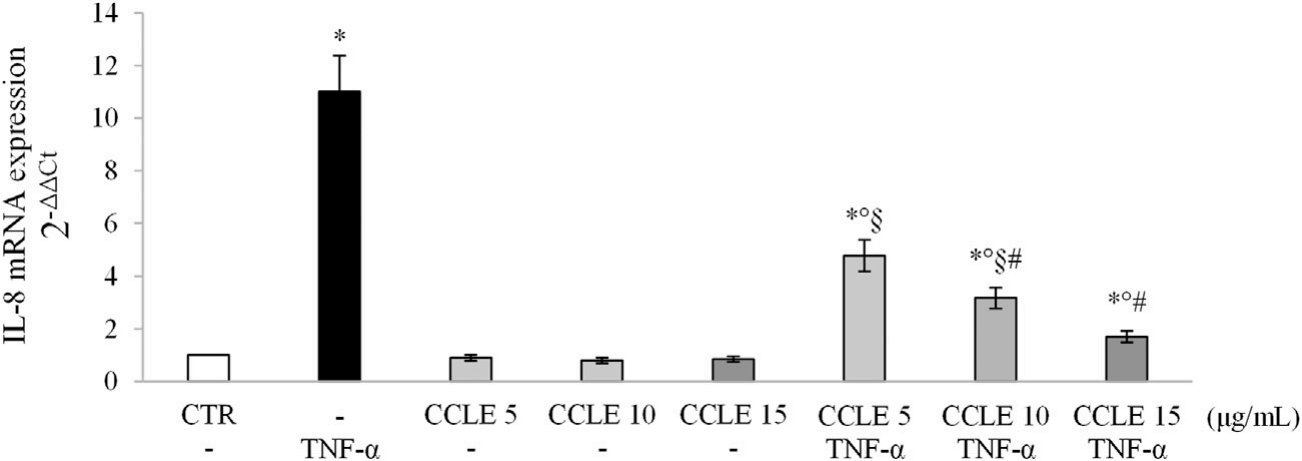
IL-8 gene expression. The Caco-2 monolayer was pretreated with CCLE (5, 10, and 15 μg/ml) for 24 h, and subsequently exposed to 50 ng/ml TNF-α for 6 h. Cultures treated with the vehicles alone were used as controls (CTR). mRNA expression levels were analyzed by real-time PCR and data were expressed as 2^−ΔΔCt^ and normalized to CTR. 18S rRNA was used as housekeeping gene. Results, deriving from three independent experiments, are reported as mean ± SD. ^*^ *p* < 0.05 vs CTR; *°p* < .05 vs TNF-α; ^§^ *p* < .05 vs all doses of CCLE alone; ^#^ *p* < .05 vs lower doses of CCLE + TNF-α.

The activation of NF-κB dimers leads to the binding of them to specific DNA sequences thus modulating the gene transcription of molecules such as pro-inflammatory cytokines (Oeckinghaus and Ghosh, 2009), and contributes to exacerbate intestinal inflammation. In order to confirm the transcriptional activity of NF-κB, IL-8 mRNA expression has been evaluated using real-time PCR. Caco-2 cells exposed to TNF-α showed an overexpression of IL-8 compared to control cells (Figure 3). On the contrary, CCLE pretreatment was able to reduce IL-8 mRNA values in a dose-dependent manner. CCLE alone showed no effect on the modulation of IL-8 gene expression.

### CCLE Inhibits COX-2 Expression Induced by TNF-α

**FIGURE 4:**
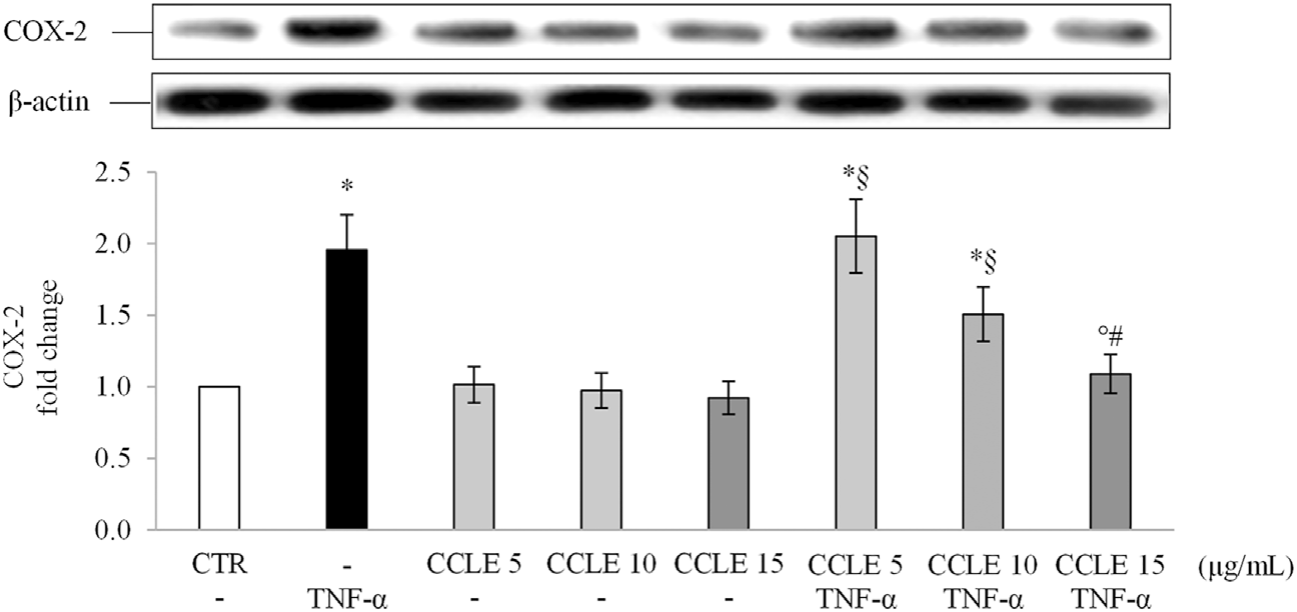
COX-2 protein expression. The Caco-2 monolayer was pretreated with CCLE (5, 10, and 15 μg/ml) for 24 h, and subsequently exposed to 50 ng/ml TNF-α for 6 h. Cultures treated with the vehicles alone were used as controls (CTR). Caco-2 whole cell lysates were analyzed by Western blot, and the expression of the COX-2 protein was evaluated. Results are reported as fold change against CTR and expressed as mean ± SD of three independent experiments. COX-2 intensity values were normalized to the corresponding β-actin value. ^*^ *p* < .05 vs CTR; °*p* < .05 vs TNF-α; ^§^ *p* < .05 vs all doses of CCLE alone; ^#^ *p* < .05 vs lower doses of CCLE + TNF-α.

COX-2 is an inducible enzyme affecting prostaglandins (PGs) and thromboxanes (TXs) production, that is involved in the pathogenesis of IBDs (Sheibanie et al., 2007). Since this enzyme is induced at inflammation sites through the NF-κB pathway (Tsatsanis et al., 2006), the intracellular concentrations of COX-2 were also evaluated. As shown in Figure 4, protein levels of COX-2 were up-regulated in Caco-2 cells following stimulation with TNF-α 50 ng/ml. When cells were pretreated with CCLE, the levels of COX-2 were significantly reduced in a dose-dependent manner. CCLE alone did not affect COX-2 levels. These results confirm once again the protective effect of CCLE against intestinal inflammation induced by TNF-α.

### Protective Effect of CCLE on the Intracellular Redox Status Alteration Induced by TNF-α

The activity of redox-sensitive NF-κB transcriptional factor is related to the intracellular redox state and its activation/nuclear translocation is stimulated by ROS (Buelna-Chontal and Zazueta, 2013), while the gene expression of cytokines regulated by NF-κB can be prevented by natural antioxidant compounds (Khan et al., 2020). Since polyphenolic compounds have been extensively shown to improve intracellular redox state (Bjørklund and Chirumbolo, 2017; Teixeira et al., 2019), we evaluated CCLE effect on TNF-α-induced oxidative stress in Caco-2 cells, by investigating the ROS levels and TAA levels, markers of the intracellular redox state. Exposure of Caco-2 cells to TNF-α caused an imbalance of the cellular redox state as demonstrated by the significantly increased ROS levels and reduced intracellular TAA levels compared to control cells (Table 1). On the contrary, CCLE has shown a protective effect against the alteration of the cellular redox state induced by TNF-α. In fact, CCLE pretreatment increased TAA values both in cells exposed and not exposed to TNF-α, and significantly reduced the intracellular production of ROS induced by TNF-α at all the tested concentrations. Moreover, the higher CCLE tested dose (15 μg/ml) significantly reduced ROS levels with respect to control cells. Furthermore, we determined the intracellular levels of GSH, an antioxidant involved in several biological functions and an important marker of the intracellular redox state (Cimino and Saija, 2008). Cell exposure to TNF-α for 6 h resulted in a significant reduction in intracellular levels of GSH. On the other hand, the pretreatment with CCLE prevented the depletion of GSH induced by TNF-α. Interestingly, CCLE was able to increase GSH levels even in cells not exposed to TNF-α.

**TABLE 1:** Intracellular redox status. The Caco-2 monolayer was pretreated with CCLE (5-15 μg/ml) for 24 h, and subsequently exposed to 50 ng/ml TNF-α for 6 h. Cultures treated with the vehicles alone were used as controls (CTR). ROS levels are reported as % DCFH-DA fluorescence intensity relative to control. Cellular GSH levels and total antioxidant activity (TAA) are reported as nmoles of GSH/mg of proteins and nmoles of trolox equivalents/mg of proteins respectively and expressed as mean ± SD of three independent experiments.

Treatments	ROS (% of control)	TAA (nmol/mg)	GSH (nmol/mg)
CTR	100 ± 4.0	38.7 ± 0.6	15.0 ± 0.9
TNF-α	119.1 ± 4.8^a^	35.1 ± 0.9^a^	11.8 ± 0.8^a^
CCLE 5 μg/ml	98.4 ± 4.1	42.3 ± 1.7^a^	15.9 ± 1.1
CCLE 10 μg/ml	90.9 ± 3.6^a^	43.1 ± 1.2^a^	17.4 ± 1.2^a^ ^,^ ^b^
CCLE 15 μg/ml	83.0 ± 3.2^a^ ^,^ ^c^	51.9 ± 1.9^a^ ^,^ ^c^	18.1 ± 1.2^a^
CCLE 5 μg/ml + TNF-α	104.8 ± 4.3^b^	43.1 ± 0.9^a^ ^,^ ^b^	13.1 ± 0.9^d^
CCLE 10 μg/ml + TNF-α	100.3 ± 5.9^b^	42.9 ± 2.3^a^ ^,^ ^b^	13.8 ± 0.9^d^
CCLE 15 μg/ml + TNF-α	85.3 ± 3.3^a^ ^,^ ^b^ ^,^ ^e^	49.5 ± 1.4^a^ ^,^ ^b^ ^,^ ^e^	14.6 ± 1.0^b^ ^,^ ^d^

a *p* < .05 vs CTR.

b *p* < .05 vs TNF-α.

c *p* < .05 vs lower doses of CCLE.

d *p* < .05 vs same dose of CCLE, alone.

e *p* < .05 vs lower doses of CCLE + TNF-α.

### CCLE Exerts its Protective Effect by Activating the Nrf2 Pathway

**FIGURE 5:**
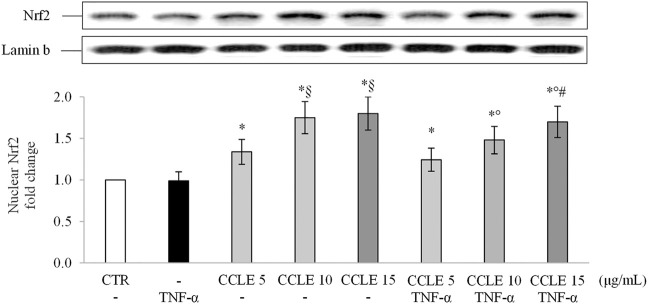
Nuclear Nrf2. The Caco-2 monolayer was pretreated with CCLE (5-15 μg/ml) for 24 h, and subsequently exposed to 50 ng/ml TNF-α for 6 h. Cultures treated with the vehicles alone were used as controls (CTR). Caco-2 nuclear lysates were analysed by Western blot, and nuclear localization of the Nrf2 protein was evaluated. Nrf2 intensity values were normalized to the corresponding Lamin B value. Results are reported as fold change against CTR and expressed as mean ± SD of three independent experiments. ^*^ *p* < .05 vs CTR; *°p* < .05 vs TNF-α; ^§^ *p* < .05 vs CCLE 5 μg/ml; ^#^ *p* < .05 vs CCLE 5 μg/ml + TNF-α.

Scientific evidence demonstrates the ability of polyphenols to modulate the endogenous antioxidant power by activating the Nrf2 pathway (Bowtell and Kelly, 2019; Zhou et al., 2019). The transcription factor Nrf2 is the master regulator of the antioxidant response (Saha et al., 2020), protecting cells from stressors that increase the production of ROS (Huang et al., 2015). Stress conditions or inducers of Nrf2 increase the nuclear accumulation of Nrf2 (Zhang and Hannink, 2003) stimulating the synthesis of endogenous antioxidant and phase II detoxifying enzymes (Forman et al., 2014; Abed et al., 2015; Mutter et al., 2015). Therefore, to determine the potential mechanism by which CCLE is able to determine its protective effects, the levels of Nrf2 in the nuclear lysates of Caco-2 cells were evaluated. In agreement with data previously reported by Ferrari and coworkers (Ferrari et al., 2016), TNF-α has no effect on the activation/translocation of Nrf2. On the contrary, CCLE pretreatment resulted in a significant increase in the nuclear levels of Nrf2, compared to control cells, both in cells exposed or not to TNF-α (Figure 5).

**FIGURE 6:**
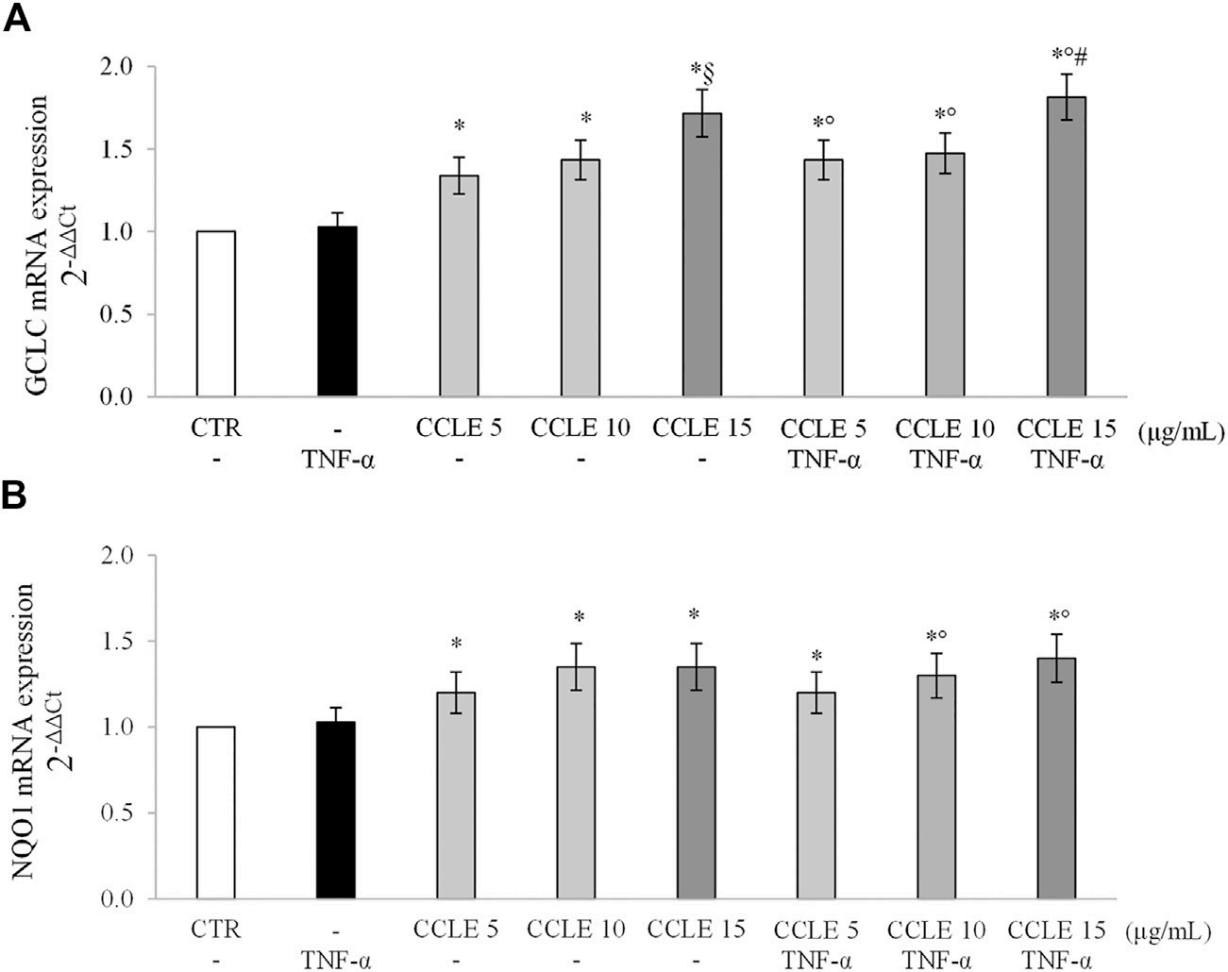
**(A)** GCLC and **(B)** NQO1 gene expression. The Caco-2 monolayer was pretreated with CCLE (5-15 μg/ml) for 24 h, and subsequently exposed to 50 ng/ml TNF-α for 6 h. Cultures treated with the vehicles alone were used as controls (CTR). mRNA expression levels were analyzed by real time PCR and data were expressed as 2^−ΔΔCt^ and normalized to CTR. 18S rRNA was used as housekeeping gene. Results, deriving from three independent experiments, are reported as mean ± SD. ^*^ *p* < .05 vs CTR; °*p* < .05 vs TNF-α; ^§^ *p* < .05 vs CCLE 5 μg/ml; ^#^ *p* < .05 vs lower doses of CCLE + TNF-α.

Glutamate-cysteine ligase (GCL) is the rate limiting enzyme that catalyzes the first step of GSH biosynthesis. It consists of two subunits, catalytic (GCLC) and modified (GCLM), whose expression is modulated by Nrf2 (Lu, 2013). NAD(P)H quinone oxidoreductase-1 (NQO1), instead, is a well-known phase I detoxifying enzyme modulated by Nrf2 (Hayes et al., 2016). Therefore, in order to confirm the ability of CCLE to activate an adaptive antioxidant response, the gene expression of GCLC and NQO1 was evaluated. As reported in Figure 6, CCLE was able to significantly increase, at all the tested concentrations, GCLC and NQO1 mRNA levels in both cells exposed or not to TNF-α, supporting the hypothesis that the protective effect of CCLE against TNF-α-induced intestinal epithelial inflammation is due to the activation of the Nrf2 pathway.

## Discussion

Hyperactivation of mucosal immune cells plays a crucial role in causing a chronic inflammatory state in IBDs. Deregulated cytokine production by immune cells triggers a strong release of proinflammatory cytokines such as TNF-α, resulting in damage to intestinal tissue. TNF-α, by binding to TNFR1 and TNFR2 receptors, initiates the inflammatory process mainly via the activation of signal pathways regulated by the transcription factor NF-κB. This transcription factor, whose expression is highly increased in patients with IBDs (McDaniel et al., 2016), has been defined as one of the main immunoregulators and its activity greatly influences the severity and course of intestinal inflammation (Atreya et al., 2008). NF-κB modulates the expression of numerous genes involved in the inflammatory process, and the cytokines produced as consequence of NF-κB activation are themselves capable of stimulating the NF-κB nuclear accumulation, ultimately leading to a perpetual inflammatory state. Natural bioactive molecules able to modulate different cell signaling pathways could be useful as new therapeutic strategies in IBDs. Numerous studies have demonstrated the benefits of polyphenols in controlling inflammation in IBDs thanks to their ability to suppress the NF-κB proinflammatory pathway (Kim et al., 2005; Nishitani et al., 2013; Chao et al., 2017; Zhang et al., 2017; Mazieiro et al., 2018; Nunes et al., 2018), and to activate the Nrf2 antioxidant signaling pathway (Ferrari et al., 2016; Li et al., 2016; Nunes et al., 2016; Serra et al., 2016; Zhou et al., 2019; Li et al., 2020).

The aim of this study was to evaluate the molecular mechanisms of the *in vitro* protective effect of CCLE against TNF-α-induced acute intestinal inflammation on differentiated Caco-2 cells. The results obtained demonstrate the ability of CCLE to protect epithelial intestinal cells against TNF-α-induced damage by inhibiting the NF-κB inflammatory pathway. In fact, nuclear levels of NF-κB (p65), as well as the activation of IKKα/β, the upstream kinase regulating NF-κB translocation/activation, was reduced by pretreatment with CCLE. NF-κB is responsible for regulating the expression of a wide variety of genes that encode for pro-inflammatory proteins which are present in high concentrations in patients with IBDs (Guan and Zhang, 2017; Leppkes and Neurath, 2020) contributing to exacerbate intestinal inflammation. IL-8 is the first modulator of the immune epithelial response since it is one of the main cytokines released by the inflamed epithelium (Eckmann et al., 1993). CCLE effects were further confirmed since the extract reduced TNF-α-induced overexpression of IL-8 mRNA levels. Additionally, COX-2 protein expression, an NF-kB inducible enzyme playing a main role in the production of inflammatory mediators, was inhibited by CCLE pretreatment.

Oxidative stress and ROS can directly or synergistically mediate an up-regulation of NF-κB activation. Indeed, activators of this pathway, such as TNF-α or IL-1β, led to increased ROS production, in part through NADPH oxidases (Pantano et al., 2006). Reduced intracellular GSH and consequent increased levels of oxidized glutathione (GSSG) were also demonstrated to mediate IkBα phosphorylation and then NF-κB activation (Fraternale et al., 2021). Since the modulation of NF-κB is related to the imbalance of the redox state, the effects of CCLE on inflammatory signaling pathway may be attributed to its ability to restore the redox status imbalance induced by TNF-α. In Caco-2 cells, pretreatment with CCLE improved intracellular redox status with a significant increase in TAA and GSH values, and a correspondent reduction of ROS levels. Furthermore, the increase in intracellular antioxidant potential exerted by CCLE support the hypothesis of an inhibitory effect of this extract on NF-κB pathway since CCLE 15 μg/ml was able to reduce NF-κB and pIKK levels below that of control cells.

Several polyphenols are known Nrf2 activators due to the capability to inhibit the Keap1-Nrf2 protein–protein interaction and to degrade Keap1 and modulate the Nrf2 related pathway (Zhou et al., 2019). In our experimental conditions, we observed that CCLE was able to improve basal intracellular antioxidant power in both TNF-α-unexposed or -exposed Caco-2 cells. These effects may be related to the ability of polyphenols to stimulate an adaptive cellular response, and thus to determine an indirect antioxidant action, by activating the Nrf2 signaling pathway (Zhou et al., 2019). Our results demonstrated that CCLE induces the nuclear translocation of Nrf2 and the expression of the Nrf2-regulated downstream genes, such as GCLC and NQO1, in Caco-2 cells exposed or not to TNF-α, so confirming that the beneficial effect of CCLE against intestinal inflammation may be mediated by an adaptive antioxidant response.

In conclusion, the data obtained in our *in vitro* study demonstrate that CCLE is able to protect epithelial intestinal cells against inflammatory stimuli. These finding are in agreement with those previously reported by Jiménez-Moreno et al. (Jiménez-Moreno et al., 2019) showing that extracts from non-edible parts of *Cynara cardunculus* subspecies *scolymus*, containing significant amounts of chlorogenic acid and luteolin glycosides, are able to overcome ROS overproduction in differentiated Caco-2 cells exposed to H_2_O_2_. Our data, for the first time, demonstrated that the effects of polyphenol rich extract from *C. cardunculus* leaves against inflammation at intestinal level are related to its capability to modulate cell signaling pathways involved in redox status control and inflammation. In fact, CCLE counteracts the acute proinflammatory effects induced by TNF-α in the intestinal epithelium suppressing NF-κB and activating Nrf2 pathways. These findings also support the hypothesis of a crosstalk between the NF-κB and Nrf2 signaling pathways which cooperate in regulation of cellular response to stress or inflammation conditions (Saha et al., 2020). The activity of this extract may be very likely related to the main polyphenols contained in it. In agreement with our suggestion, chlorogenic acid isomers activate Nrf2 signaling and were shown to alleviate IL-8 production and increase GSH levels in differentiated Caco-2 cells exposed to human interferon γ (IFNγ) and phorbol myristate acetate (Liang and Kitts, 2018b; a; Liang et al., 2019). Furthermore, luteolin can reduce damage induced by TNF-α and IFNγ in Caco-2 cells via inactivation of STAT3 signaling pathway (Li et al., 2021), and attenuates ethanol-induced injury by inhibiting MAPK/NF-κB/MLCK pathway and upregulating Nrf2 Pathway in Caco-2 monolayers (Yuan et al., 2021).

In conclusion, our data clearly evidence that, although considered a waste, *Cynara cardunculus* leaves may be used to obtain extracts rich in bioactive polyphenols potentially useful for prevention and treatment of inflammatory intestinal diseases.

## Data Availability

The original contributions presented in the study are included in the article/Supplementary Material, further inquiries can be directed to the corresponding author.
